# Carboxydotrophic Acetogenesis in Alkaline Conditions Results in Transient Formate Production by the Halo‐Alkaliphilic Acetogen *Haloacetibacter carboxydivorans* Gen. Nov. sp. Nov

**DOI:** 10.1111/1758-2229.70254

**Published:** 2026-01-27

**Authors:** Martijn Diender, Isabelle M. Keijsers, Anastasia Galani, Timo van Roosmalen, Alfons J. M. Stams, Diana Z. Sousa

**Affiliations:** ^1^ Wageningen University & Research Wageningen the Netherlands; ^2^ Centre for Living Technologies, EWUU Alliance Utrecht the Netherlands

**Keywords:** acetogenesis, formate dehydrogenase, soda lake, synthesis gas, wood‐Ljungdahl pathway

## Abstract

Carboxydotrophic acetogens are found widespread in the environment, yet the strains characterised to date are almost exclusively mild acidophiles or neutrophiles, often isolated from gut or freshwater systems. Here, we describe a novel carboxydotrophic halo‐alkaliphilic, acetogenic bacterium, strain MD4, isolated from a CO‐fed bioreactor operated under high salt and alkaline conditions. Phylogenetic analysis suggests that strain MD4 is the first representative of a novel genus, branching between the Alkalibacter and Alkalibaculum genera, for which we propose the name *Haloacetibacter carboxydivorans*. The bacterium tolerates a wide range of sodium (0.01–2.5 M) and pH (7–10), but was not exceptionally tolerant to metals such as copper, nickel and cobalt. During growth on CO, strain MD4 produced formate and acetate, the former being co‐consumed upon low CO availability to drive acetogenesis. Interestingly, common by‐products of carboxydotrophic acetogenesis—ethanol or hydrogen—were not produced, suggesting that formate production may serve as a form of redox homeostasis during alkaliphilic carboxydotrophy. Genome analyses revealed no clear bifurcating formate dehydrogenase or formate hydrogen lyase, but during carboxydotrophy the transcriptome showed high expression of two putative bifurcating hydrogenases, and a NADH‐dependent formate dehydrogenase, potentially playing a role in the dynamic formate metabolism.

## Graphical abstract

This work presents the physiological exploration of the halo‐alkaliphilic, carboxydotrophic acetogen bacterium MD4. In contrast to well‐characterised neutrophilic and slightly acidophilic acetogenic carboxydotrophs, this work shows an important role for formate production, instead of ethanol, as a redox exhaust mechanism in alkaline conditions.

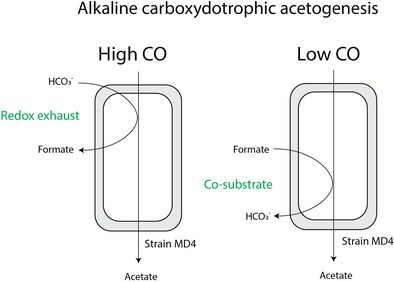

## 1 Introduction

The anaerobic conversion of CO by methanogens, acetogens and hydrogenogens has been widely reported (Diender et al. 2015; Robb and Techtmann 2018). Additionally, the gene relevant for anaerobic carboxydotrophy, carbon monoxide dehydrogenase (CODH), is found widespread in (meta)genomes of bacteria and archaea (Inoue et al. 2019; Techtmann et al. 2012), pointing towards a broad occurrence of carboxydotrophs in nature. However, most of the so far isolated carboxydotrophic acetogens derive from the gut or freshwater environments (Diender et al. 2015; Poehlein et al. 2025), with many other environments remaining unexplored. Well‐studied acetogenic species, such as *Acetobacterium woodii* (Bache and Pfennig 1981) and *Clostridium autoethanogenum* (Abrini et al. 1994), grow optimally in a pH range of 4–7, limiting our understanding of acetogenic carboxydotrophy to neutral and mildly acidic conditions. Exploring underrepresented environments for the isolation of carboxydotrophic acetogens may reveal distinct physiological capabilities, and provide a foundation for the discovery of novel biocatalysts.

Halo‐alkaline systems—characterised by the presence of high levels of sodium carbonate/bicarbonate salts and elevated pH (Jones et al. 1977)—are among the relatively underexplored environments for acetogenic carboxydotrophy. Despite double extremophilic conditions (high salinity and pH), a wide range of microbial physiologies is found in these environments, both fermentative and respiratory (Sorokin and Kuenen 2005; Uma et al. 2020). Currently there are only two isolates known to perform carboxydotrophic acetogenesis above a salinity of 1 M sodium and pH > 8: *Alkalibacter mobilis* (Khomyakova et al. 2021) and *Natranaerofaba carboxydovora* (Sorokin et al. 2021).

Most well‐studied carboxydotrophic acetogens, such as *C. autoethanogenum*, convert CO into acetate and ethanol. Ethanol production is promoted under low pH, high acetic acid concentrations and abundant reducing power from CO metabolism (Richter et al. 2016). In contrast, alkaline environments are characterised by low proton concentration, which limits proton availability and causes organic acids to predominantly exist in their dissociated forms. These environments likely pose a physiological challenge to carboxydotrophic acetogens, as key redox‐balancing reactions—such as proton reduction to hydrogen and conversion of acetic acid to ethanol—become energetically less favourable.

This study reports on the isolation and characterisation of a novel halo‐alkaliphilic carboxydotrophic acetogen, strain MD4, isolated from sludge of a CO‐fed bioreactor operated at high salt and alkaline conditions. The physiological characterisation of strain MD4 shows its capability to grow acetogenically on CO, producing acetate and formate as its major products. Despite that no known bifurcating formate dehydrogenase, nor formate hydrogen lyase, could be identified from the genome, we hypothesize that the strongly upregulated bifurcating hydrogenases in combination with the NADH‐dependent formate dehydrogenase play a role in formate metabolism. Strain MD4 represents a novel species of a novel genus, for which the name *Haloacetibacter carboxydivorans* gen. nov. sp. nov is proposed.

## 2 Experimental Procedures

### 2.1 Medium and Microbial Cultivation

The enrichment and isolation of strain MD4 was performed in carbonate buffered medium with a pH set around 9 (0.3 M Na_2_CO_3_, 0.8 M NaHCO_3_, 5.7 mM KH_2_PO_4_, 10 mM KHCO_3_, 5 mM NH_4_Cl, 0.1 mM CaCl_2_, and 0.5 mM MgCl_2_). Trace elements were added as 1 mL/L solution (containing per litre: 1000 mg Sodium EDTA, 370 mg FeCl_2_·4 H_2_O, 60 mg H_3_BO_3_, 26 mg MnCl_2_·2 H_2_O, 40 mg CoCl_2_·6 H_2_O, 10 mg ZnCl_2_, 3 mg CuCl_2_, 32 mg KAl(SO_4_)_2_·12H_2_O, 31 mg NiCl_2_·6H_2_O, 40 mg NaOH, 10 mg Na_2_SiO_3_·5H_2_O, 10 mg Na_2_MoO_4_·2H_2_O, 10 mg Na_2_SeO_3_·5H_2_O, and 10 mg Na_2_WO_4_·2H_2_O). Cultivation was done in sealed glass bottles (total volume 121 mL or 250 mL) with 50 mL medium. The bottle headspace was replaced with nitrogen gas, and composition was further adjusted with the desired gases (e.g., CO and H_2_) by manually removing/adding gas with a 50 mL syringe. The medium was autoclaved and, when required, supplemented with components such as yeast extract (0.1 g/L) and reducing agent (either 1 mM sodium sulfide or 2 drops of 100 mM titanium citrate), from sterilised anaerobic stock solutions. For physiological tests and reactor cultivation Na_2_CO_3_ was omitted from the medium, 10 mM KHCO_3_ was replaced by 10 mM KCl, and pH was set to 8.5 using 3 M NaOH or 3 M HCl.

For growth in bioreactors a 1.5 L (total volume) Applikon reactor (Getinge, Göteborg, Sweden) was operated in batch mode. Gases CO and N_2_ were supplied using mass flow controllers (Brooks Instruments BV, Ede, the Netherlands). The liquid volume in the reactors was set to 750 mL. The pH was maintained at a value of 9 using 3 M KOH. Sterilised reactors were connected to the control tower, initiating temperature (37°C) and pH control. Prior to inoculation, reactors were flushed for 3 h with N_2_ at a rate of 20 mL/min. Yeast extract, and sulfide were introduced in the reactor in the same concentration as described for bottle cultivation. Before inoculation the gas flow was switched to 1 mL/min CO plus 1 mL/min N_2_. Redox and pH were tracked using an Applisens online redox/pH probe (Getinge, Göteborg, Sweden). After reducing the medium below −300 mV the reactor was inoculated with the exponentially growing culture in a 1:20 ratio.

### 2.2 Source of Inoculum and Microbial Isolation

Anoxic sludge (40 mL) was withdrawn from a continuously operated lab‐scale bioreactor operating at a pH of 9 and 1.5 M sodium, treating an influent containing 25 mM sulfate and 12.5 mM thiosulfate with CO and H_2_ as substrate (Plugge et al. 2020; Sousa et al. 2015). The culture enrichment was set up with 1 mL of the anoxic sludge sample in 50 mL medium, under a partial pressure of 0.4 bar CO and 1 bar N_2_. Culture was incubated shaking at 150 rpm, 37°C and in the dark. Upon CO depletion, the enrichment was transferred to new bottles in serial dilution, ranging from 10^−2^ to 10^−10^. The most diluted grown culture was used to inoculate the next series. After three rounds of serial dilution, the enrichment was used to inoculate soft‐agar bottles containing halo alkaline medium with 5 g/L agar. Agar solution and medium were prepared at double concentration under a nitrogen headspace and autoclaved separately. After autoclaving, both bottles were kept at 65°C to prevent agar from solidifying. Required additives and reducing agents were added to an empty rubber‐capped bottle, containing a headspace with 0.4 bar CO partial pressure. The concentrated agar and medium were simultaneously injected (each 10 mL) into the prepared bottles. 1 mL of the enrichment sample was added immediately after. Bottles were gently swirled to mix the inoculum and medium components and put on ice for fast solidification. Solidification was done under an angle of 45° to prevent water from covering the gel when stored upright. Soft‐agar bottles were incubated at 37°C in the dark, in an upright position. Individual colonies were picked from the agar and inoculated in freshly prepared liquid medium with 0.4 bar CO in the presence of 0.1 g/L yeast extract. Out of the 5 colonies picked, only one culture picked up growth, resulting in the isolation of strain MD4. Purity of the strain was assessed using 16S rRNA gene sequencing (Macrogen Europe, the Netherlands) and microscopic analysis. Strain MD4 is deposited in the DSMZ (Germany) culture collection (DSM 119686) and the JCM (Japan) culture collection (JCM 39617).

### 2.3 Strain Characterisation

Unless mentioned otherwise characterisation experiments were conducted in sealed glass vials (121 mL), using glucose as a substrate. The viability range for temperature was tested from 5°C to 90°C with 5 degree intervals, and pH was tested from 6.5 to 10 with 0.5 points interval. Substrates: Glucose, Arabinose, Cellobiose, Dulcitol, Fructose, Sorbose, Sucrose, Mannose, Ribose, Xylose, Mannitol, Galactose, Peptone, Tryptone, Yeast extract, Propanol, Acetate, Formate, Lactate, Butanol, Glycerol, Methanol, Pyruvate, CO, H_2_/CO_2_ were tested for fermentative growth of strain MD4. Additionally, oxygen (1%, 5%, 10% and 21%), nitrate (10 mM), sulfate (10 mM), thiosulfate (10 mM), iron(III)citrate (10 mM), manganese(IV)oxide (0.045 g solids per bottle with 50 mL liquid) and fumarate (10 mM), were tested as electron acceptors.

For sodium concentration and metal tolerance tests, anaerobic cultivation in 96‐well plates was done. Cultivation medium was prepared as described above in glass bottles and autoclaved. Anaerobic medium was completed, with the exception of the addition of a reducing agent. Medium bottles, an actively growing culture bottle and required stock solutions of trace metals (CuCl_2_, NiCl_2_ or CoCl_2_), bicarbonate and NaCl, were brought into an anaerobic tent (Coy, Michigan, USA), and content transferred to a liquid handler (EpMotion 5070 Eppendorf, Germany). Medium was filled out in 96‐well plates and salt, carbonate and metal concentrations were altered in each well to create the desired conditions. After inoculation, plates were covered with breathe easy film (Diversified Biotech, USA), and stored in anaerobic containers at 37°C. Bicarbonate and sodium tests were performed on glucose, and each condition was generated in triplicate. For the trace metal tests each condition was generated in 8‐fold. The plates were stored in anaerobic boxes, of which the headspace was replaced by 0.8 bar CO total pressure. Start and final OD600 were measured using a plate reader (BioTek Synergy H1, Agilent Technologies, USA) situated in the anaerobic tent.

### 2.4 Analytical Techniques

Soluble compounds (organic acids, alcohols) were analysed by high pressure liquid chromatography (HPLC) using a MetaCarb 67H column (Agilent Technologies, Santa Clara, CA). The column was kept at 45°C, with H_2_SO_4_ 0.01 N as eluent at a flow rate of 0.8 mL/min. Metabolites were quantified using a UV and RI detector. Sample preparation for HPLC analysis consisted of centrifugation at 13,000×*g*, and subsequent mixing with 10 mM DMSO in 0.1 N H_2_SO_4_ in a 2:3 ratio. Concentrations below 0.1 mM could not be accurately quantified and are referred to as trace amounts.

For amino acid analysis: 10 μL sample supernatant, 100 μL 0.15 M NaHCO_3_ (pH 9.0), and 200 μL freshly made dabsyl chloride (1.3 mg/mL in acetonitrile) were mixed and heated at 70°C for 20 min. After cooling down, 690 μL of 70% ethanol was added. Measurement was performed on a Shimadzu Prominence‐1, LC2030C‐Plus_2_ELSD HPLC (Shimadzu Benelux), equipped with an Agilent Poroshell 120 EC‐C18 column (100 × 3.0 mm, P.N. 695975‐302; Agilent Technologies Netherlands B.V.). The eluent was a gradient of water, 100 mM ammonium acetate, isopropanol, and acetonitrile. At the start, the water/ammonium acetate/isopropanol/acetonitrile ratio was 60/20/10/10, adjusted with a gradient with increasing slope to 22.5/7.5/35/35 at 18 min, and brought back to the starting gradient after 22 min. The flow was 0.45 mL/min, the column temperature 40°C, and the injection volume 5 μL. Detection was done with a UV/VIS detector at a wavelength of 436 nm.

Concentrations of sulfate, thiosulfate and nitrate were measured by anion exchange chromatography (IC) on a Dionex ICS‐2100 (Dionex, Sunnyvale, CA, USA) equipped with a Dionex IonPac AS19 column (Dionex) operated at 30°C. As eluent, KOH 22% (wt/vol) was used in a gradient ranging from 10 to 40 mM at a flow rate of 0.4 mL/min.

Gaseous compounds (H_2_, CO, and CO_2_) were analysed by injecting 0.2 mL of headspace gas in a Compact GC 4.0 (Global Analyser Solutions, The Netherlands), equipped with two columns: a molsieve 5A column operated at 100°C, for H_2_ and CO analysis; and, a Rt‐Q‐BOND column operated at 80°C, for CO2 analysis. Detection was in all cases done with a thermal conductivity detector with a detection limit of ~500 ppm.

Solubilised copper(II), iron(II)/iron(III) and manganese(II) concentrations were determined using Spectroquant kits nr. 1.14767.0001, 1.00796.0001 and 1.14770.0001 respectively (Merck, Germany).

Lipid analysis was performed by growing cultures on glucose, and the biomass pellet was sent for analysis to the DSMZ (Braunsweig, Germany).

For light microscopy a Zeiss Ax10 was used (Zeiss, Germany). For scanning electron microscopy a Zeiss Auriga system was used (Zeiss, Germany), equipped with an energy dispersive X‐ray spectroscopy system (Oxford Instruments, UK). Before analysis via electron microscopy samples were dehydrated by washing with 70% and 100% ethanol, and coated with tungsten.

### 2.5 Genomic and Transcriptomics Analyses

Genomic DNA was obtained from a 50 mL culture (250 mL bottles) grown on 0.4 bar CO. Cells were spun down and washed with 0.4 M NaHCO_3_, followed by extraction using the Gram‐positive DNA extraction kit from Epicentre (Hilden, Germany), according to the manufacturer's instructions. PacBio sequencing was done on a PacBio RS II platform (Pacific Biosciences, Menlo Park, CA). PacBio reads were assembled with Falcon v.0.3 (Chin et al. 2016). The quality of the generated assembly was assessed with QUAST v5.0.2 (Manchanda et al. 2020). The completeness and contamination of the genome were calculated with CheckM v1.1.2 (Parks et al. 2015). The genome was annotated and analyzed using RAST (Aziz et al. 2008; Overbeek et al. 2014), and additionally via the EggNOG mapper (version 2.1.12) (Cantalapiedra et al. 2021). The genome of strain MD4 was deposited in GenBank: CP174120.

For transcriptome analysis, RNA was isolated from 50 mL exponentially growing cultures (250 mL bottles) of strain MD‐4, supplied with 0.4 bar partial pressure CO (*n* = 3). Samples were rapidly cooled down on ice, and biomass pelleted via centrifugation at 4°C (5 min, 13,000×*g*), followed by immediate processing. RNA was co‐extracted with DNA (as described above); before the RNAse addition step a part of the sample was transferred to a liquid handler to further perform RNA extraction and purification using the Maxwell SimplyRNA cells kit (Promega, Madison, WA). RNA sequencing was performed by GenomeScan B.V (Leiden, the Netherlands) on the Illumina Novoseq 6000 platform yielding paired‐end reads of ~150 bp. Raw reads were trimmed using bbduk.sh of BBmap (v38.84), followed by fastQC quality check (v0.11.9). Sequences were then mapped using bbsplit.sh against protein coding sequences obtained from the RAST annotated genome. Mapped genes were counted using SAMtools view (version 1.10) (Danecek et al. 2021). Transcriptome analysis data is provided in Supporting Information.

Phylogenetic placement of strain MD4 was inferred based on a set of 120 bacterial single‐copy marker protein sequences identified in the genome and aligned using the GTDB‐Tk v1.5.0 (Chaumeil et al. 2020). Subsequently, the concatenated alignment was trimmed with trimAl v1.4.1 (‐gappyout) (Capella‐Gutiérrez et al. 2009). A maximum‐likelihood tree was generated from the trimmed alignment (5030 positions) with IQ‐TREE v2.0.6 (Nguyen et al. 2015) using extended model selection (‐m MFP) (Kalyaanamoorthy et al. 2017). The best‐fit substitution model was LG + F + R6 and the final tree was created with 1000 standard nonparametric bootstrap replicates. Digital DNA–DNA hybridization (dDDH) with closest relatives was done using the Type Strain Genome Server (TYGS) of the DSMZ (Meier‐Kolthoff and Göker 2019). Average amino acid identity (AAI) was done using the AAI‐profiler (Medlar et al. 2018).

## 3 Results

### 3.1 Enrichment and Isolation of Strain MD4

A CO‐oxidising enrichment was obtained from a CO‐fed bioreactor (Plugge et al. 2020) that was operated at a pH of 9, 1.5 M sodium, and 1 M carbonate concentration. After initial enrichment on 0.4 bar CO, acetate, formate and methane production was observed. In three subsequent rounds of dilution series on CO as sole substrate, methanogenesis was no longer detected, while acetate and formate production were still observed. Two distinct rod‐shaped microbes could be observed under the microscope, but further serial dilution of the enrichment did not result in the segregation of the two morphotypes. By making use of growth on solid media under CO headspace, small (~0.5 mm in diameter), transparent colonies became visible after 2 weeks. Picking colonies and transferring them to liquid medium, with yeast extract (0.1 g/L), and CO as substrate, resulted in the successful isolation of strain MD4.

### 3.2 Phylogeny of Strain MD4

**FIGURE 1:**
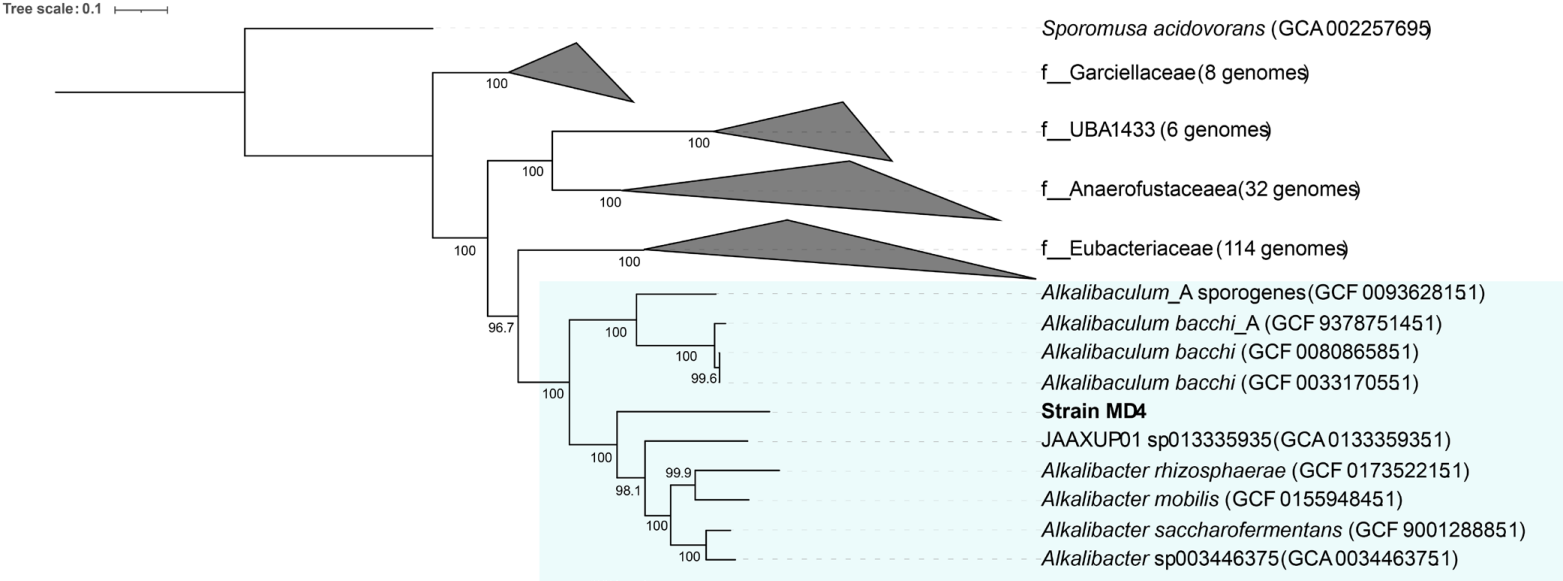
Whole genome taxonomy of strain MD4 using 169 *Eubacteriales* genomes, based on 120 marker genes.

Based on 16S rRNA gene sequence identity, the closest relative of strain MD4 is *Alkalibacter mobilis* (92.6%), a carboxydotrophic acetogen isolated from a coastal lake in Russia (Khomyakova et al. 2021). Other close relatives are *Alkalibacter saccharofermentans* (Garnova et al. 2004) and *Alkalibacter rhizosphaerae* (Namirimu et al. 2022), with 16S rRNA gene identities around 92% as well. Whole genome‐based taxonomy, using 169 genomes in the *Eubacteriales* order, resulted in the placement of strain MD4 between the *Alkalibaculum* and *Alkalibacter* genera (Figure 1). Average amino acid identity (AAI) analysis against the Uniprot database indicated that strain MD4 is most similar to *A. saccharofermentans* (71%) and *Alkalibacter rhizosphaerae* (70%), however only having a matching protein fraction against these species of about 25% (Figure S1). In silico DNA–DNA hybridization (dDDH) of the genome of strain MD4, showed the highest dDDH similarity (d_4_ value: proportion of sequence identity within the homologous parts of the underlying genomes) against *Alkalibaculum bacchi* (36.3%), and values of 18%–20% against members of the genus *Alkalibacter*. These analyses suggest significant dissimilarity on genomic, as well as on proteome levels of strain MD4 compared to other known isolates.

### 3.3 Physiology and Morphology of Strain MD4

**FIGURE 2:**
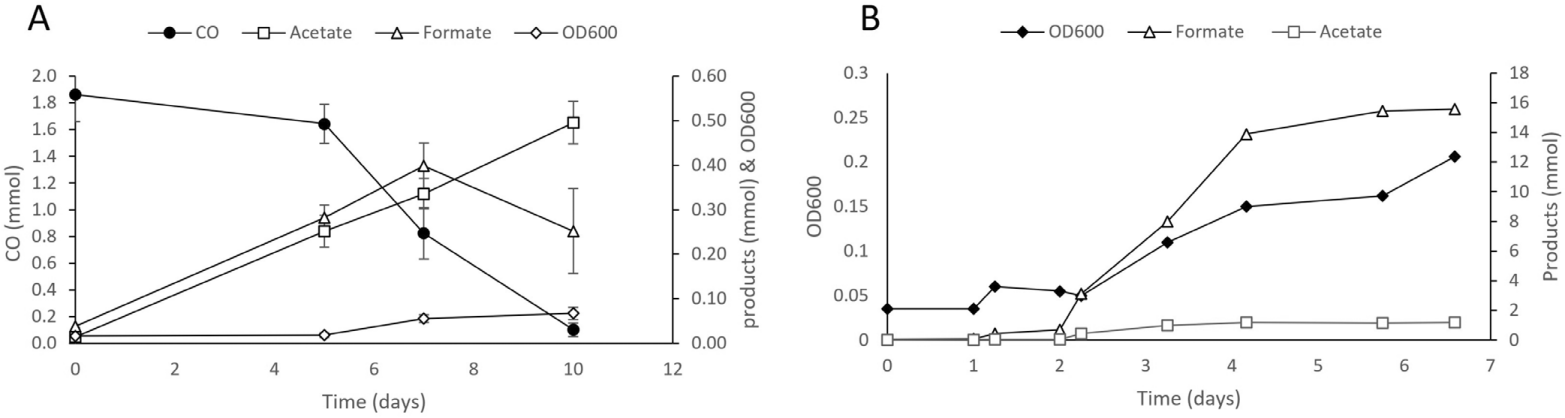
Physiological growth profile of strain MD4 displaying growth (OD600), and total amount (mmol) of products produced, during carboxydotrophic growth in (A) batch bottle (*n* = 12), error bars represent standard deviation, and (B) a CO fed bioreactor. Open symbols indicate left axis while closed symbols refer to the right axis.

Strain MD4 could successfully be cultivated on CO, and after adaptation over several transfers could grow with a CO headspace up to 150 kPa CO (maximum tested). The bacterium grows fermentatively on a range of sugars, but not on alcohols, fatty acids, other C1 substrates or H_2_/CO_2_ (Table 1). Oxygen, nitrate, sulfate, thiosulfate, iron(III) citrate, manganese(IV) oxide, and fumarate were tested as electron acceptors, but were not reduced. Growth on all tested substrates required the addition of (0.1 g/L) yeast extract. Fermentative growth on sugars results in acetate and formate as major end‐products, while occasionally forming a minor amount of succinate. During carboxydotrophic growth, acetate and formate were formed as sole fermentative end‐products. Measurement of amino acid levels in the medium after growth on CO indicated a clear presence of low (< 1 mM), but significant, concentrations of glycine, while other amino acids were not detected. Production of hydrogen and/or ethanol was not observed in any of the conditions. While formate is produced during initial carboxydotrophic growth, it is partially co‐consumed when CO reaches low levels (Figure 2A), and is not further consumed after CO is depleted. Total carbon in acetate and formate makes up for about 67% ± 6% of the carbon consumed in the form of CO. The remaining fraction of the carbon is likely in CO_2_, but could not be accurately determined due to the high carbonate background of the carbonated medium. To further study the carboxydotrophic physiology of strain MD4, growth was monitored in a pH‐controlled batch bioreactor system, fed with continuous CO flow (Figure 2B). After 3 days of growth, metabolite levels reached ~16 mmol formate and ~1 mmol acetate in the reactor, significantly exceeding the ~1:1 formate:acetate ratio observed in batch bottles (Figure 2A). In contrast to the batch incubations, no formate consumption occurred in the bioreactor, suggesting that continuous CO availability stimulated formate production.

**TABLE 1:** General characteristics and substrate utilisation by strain MD4 compared to closest related members of *Alkalibacter* and *Alkalibaculum* genera, and the more taxonomically distant halo‐alkaliphilic carboxydotroph *N. carboxydivora*.

	Strain MD4 (This work)	*Alkalibacter mobilis* (Khomyakova et al. 2021)	*Alkalibaculum bacci* (Allen et al. 2010)	*Natranaerofaba carboxydovora* (Sorokin et al. 2021)
Gram stain	Positive	n.d.	Variable	n.d.
Cell length	1–5 μm	1.6–2.1 μm	1.5–2.2 μm	2–5 μm
Cell shape	Rods	Short rods	Short rods	Bean shaped
pH range (optimum)	7–10 (8–9)	5.5–10 (8)	6.5–10.5 opt (8)	9–10.5 (9.5)
Temperature range (optimum)	15°C–40°C (35–37)	14°C–42°C (30)	15°C–40°C (37)	35°C–56°C (50)
Sodium range (optimum)	0.01–2.5 M (0.8–1.2 M)	0.2–2 M (0.3 M)	0.06 M	2.5–4.5 M (4 M)
Fermentation products on CO	Acetate, formate	Acetate, ethanol	Acetate, ethanol	Acetate, formate
GC content	38.4%	39.1%	34.0%	35.3%
Substrate utilisation				
Glucose	+	+	+	−
Arabinose	−	+	−	−
Cellobiose	+	−	−	−
Dulcitol	−	−	−	−
Fructose	+	+	+	−
Sorbose	+	−	n.d.	−
Sucrose	+	+	−	−
Mannose	+	+	+	−
Ribose	+	+	+	−
Xylose	−	−	−	−
Mannitol	+	−	−	−
Galactose	+	−	−	−
Peptone	+	+	−	n.d.
Tryptone	+	n.d.	−	n.d.
Yeast extract	+	+	−	n.d.
Propanol	−	n.d.	+	−
Acetate	−	n.d.	−	−
Formate	−	n.d.	−	−
Lactate	−	n.d.	−	−
Butanol	−	n.d.	+	−
Glycerol	−	n.d.	−	n.d.
Methanol	−	n.d.	n.d.	−
Pyruvate	−	n.d.	+	+
CO	+	+	+	+
H_2_/CO_2_	−	n.d.	+	−

Abbreviation: n.d. indicates not determined.

**FIGURE 3:**
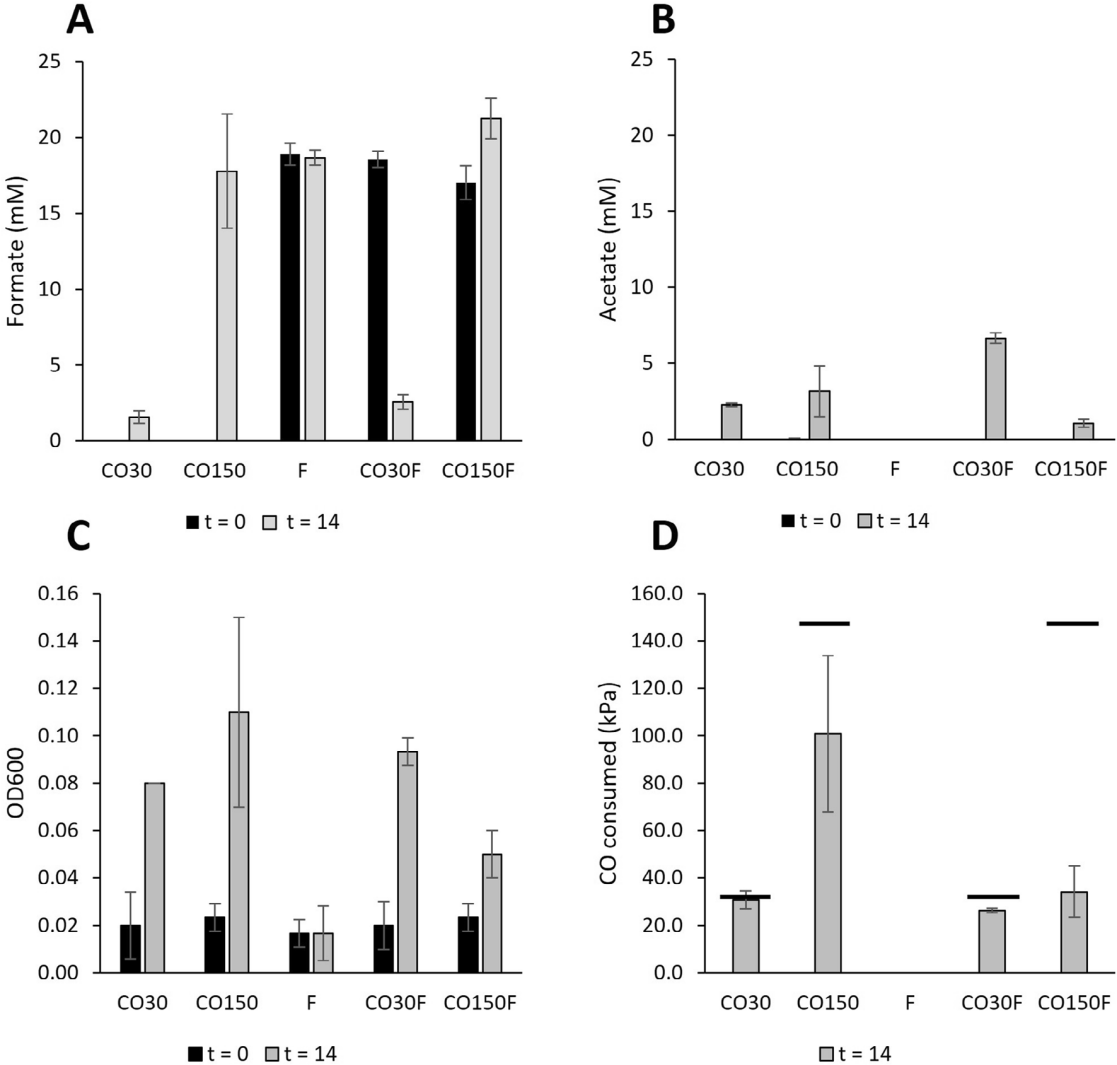
Effect of low (30 kPa) or high (150 kPa) CO, in absence or presence (20 mM) of formate, on the growth of strain MD4. Formate concentrations (A), acetate concentrations (B), OD600 (C) and CO consumed (D) are shown. For panel D, horizontal bars above the bar graphs indicate the initial amount of CO added. Standard deviations are shown over triplicates (*n* = 3), with exception of the CO30 condition, which was a duplicate (*n* = 2).

To further assess the interplay of CO availability, formate production and growth, MD4 was grown in the presence or absence of formate (20 mM) in high (150 kPa) and low (30 kPa) starting CO pressure (Figure 3). After 14 days, all cultures had seized growth. Cultures without initially added formate resulted in the production of acetate and formate, significantly producing more formate at higher CO pressure (Figure 3A). In the presence of 20 mM initial formate, at low CO pressure, the formate was almost fully consumed, leading to significantly more acetate production (Figure 3B), while at high CO pressure additional formate was produced on top of the initial formate (Figure 3A). In the presence of formate and absence of CO, no microbial activity was observed, confirming earlier results that formate cannot be used as the sole substrate by MD4. Conditions with low CO showed slightly more growth in the condition with formate added (Figure 3C). The presence of initial formate at high CO, showed little growth, indicating inhibitory effects of initial formate at high CO pressures (Figure 3C). Cultures with low CO completely depleted the originally added CO, while in all the high CO bottles microbial activity stagnated before finishing the added CO, showing least consumption when initial formate was present (Figure 3D).

**FIGURE 4:**
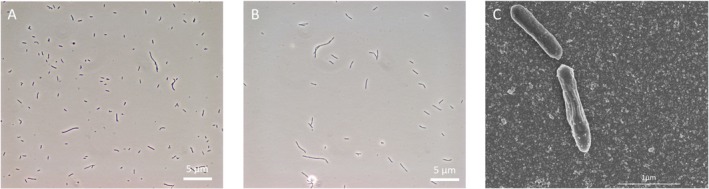
Microscopic observation of strain MD4 grown on (A) glucose and (B) CO via light microscopy, and (C) CO‐grown cells via scanning electron microscopy.

Strain MD4 grows as rod‐shaped cells that vary in length from 1 to 5 μm, which occasionally can become much longer (Figure 4). Strongly elongated cells become more present near the end of growth, and in some cases show division septa, resulting in chained cell morphology (Figure 4A,B). Cells stained Gram‐positive, were non‐motile and grew suspended when cultivated in liquid medium, irrespective of the substrate used. Membrane lipid composition consisted mainly of a variety of C16 and C18 ester‐bound alkyl chains (Table S1). Additionally, a significant amount of C16 (~4%) and C18 (~12%) dimethyl acetals, derived from alk‐1‐enyl ether substituents of plasmalogen lipids, were detected.

### 3.4 Effect of Environmental Factors on Growth and Metal Tolerance

**FIGURE 5:**
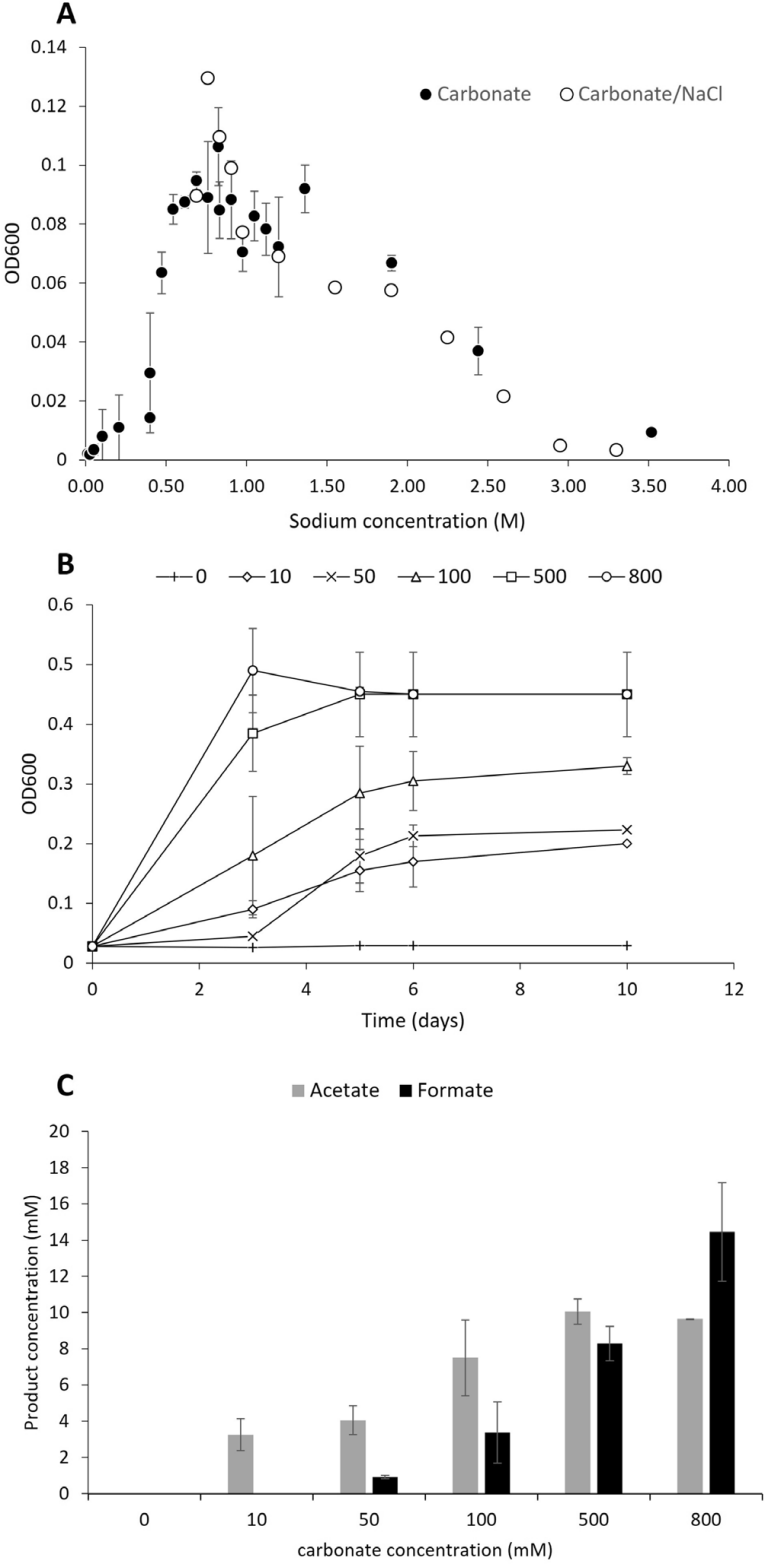
Growth of strain MD4 on glucose at various carbonate and sodium concentrations. (A) Growth performed in 96‐wells plates at different sodium (bi)carbonate concentration (solid dots) and at different NaCl concentration with a background of 0.8 M sodium bicarbonate (open dots) (*n* = 3). (B) Growth profile (OD600) of strain MD4 at different concentrations of bicarbonate (mM). (C) The final product spectrum of strain MD4 in the different bicarbonate incubations shown in panel B.

Strain MD4 grows in a temperature range from 15°C to 40°C, with an optimum around 35°C–37°C (Figure S2). Growth occurred in a range of pH 7–10 with a broad optimum around 8–9 (Figure S2). At temperatures above 45°C, and at pH higher than 9.5, strong Maillard reaction colouring was observed in the medium interfering with turbidity analysis. In these cases, the presence/absence of growth was additionally checked via light microscopy, confirming the absence of growth above 45°C and at pH 10. The bacterium performs optimally under strict anaerobic conditions, but could tolerate up to 1% oxygen in the headspace when grown on glucose. Strain MD4 grows in a broad range of sodium (bi)carbonate concentrations, from 0.01 M up to 2.5–3 M (with an optimum at 0.8–1.2 M) (Figure 5A). Tests with a background of 0.8 M sodium bicarbonate and increasing sodium concentration with NaCl (up to 3.5 M sodium) provided similar results, with growth observed up to 2.5–3 M of sodium (Figure 5A). To test the effect of decreasing bicarbonate concentrations below 0.8 M, strain MD4 was grown on glucose with different NaCl/bicarbonate ratios, keeping sodium concentration (0.8 M) and initial pH (~8.5) similar (Figure 5B). The lowest carbonate concentration tested at which growth was observed was 0.01 M, but was significantly slower compared to 0.8 M carbonate (Figure 5B). With increasing carbonate concentrations, the fermentation spectrum on glucose strongly shifted towards formate production (Figure 5C).

**FIGURE 6:**
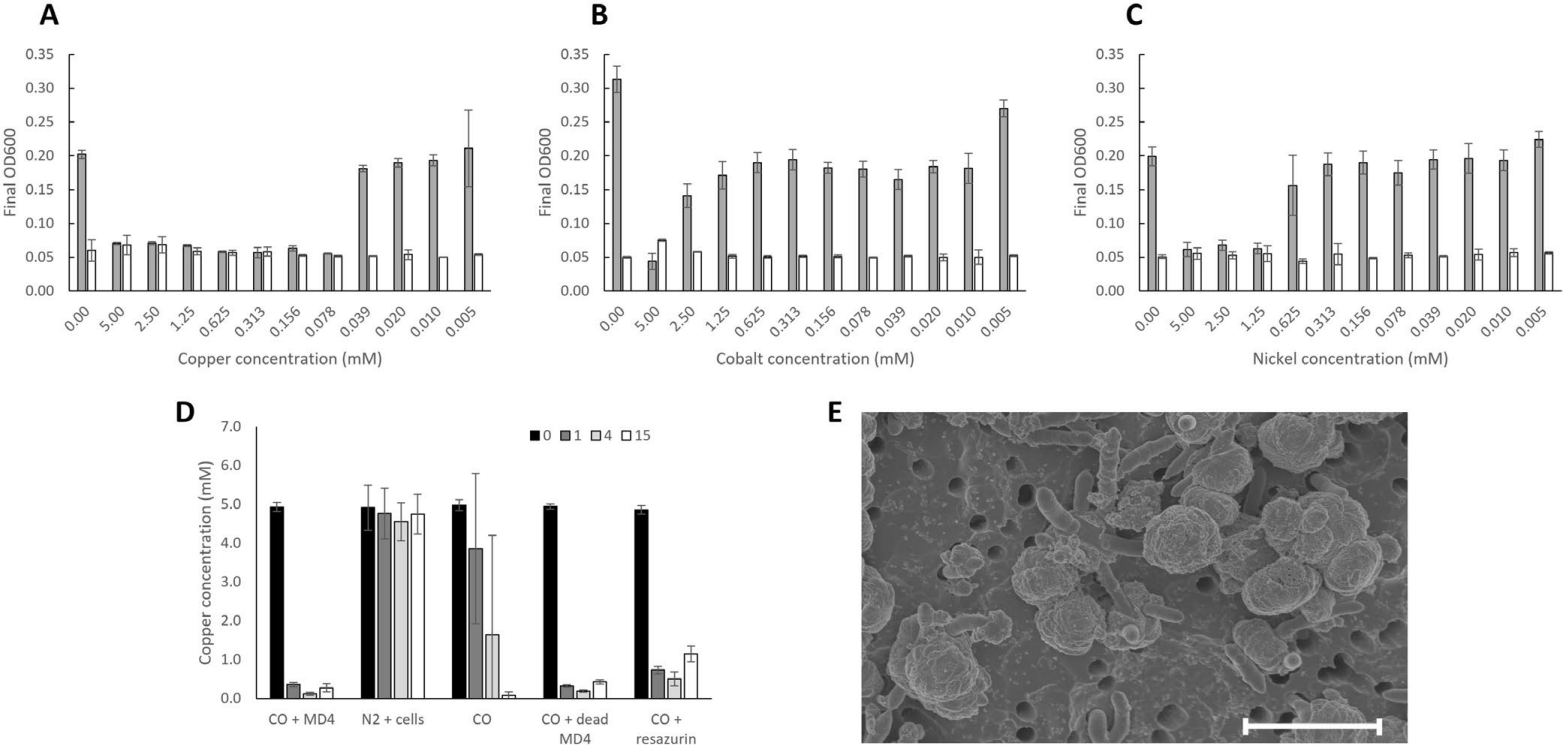
Carboxydotrophic growth of strain MD4 in presence of trace metals copper, nickel and cobalt. Growth was tested in presence of a range of (A) CuCl_2_, (B) CoCl_2_ and (C) NiCl_2_ (*n* = 8). Shaded bars indicate biotic test, while white bars represent abiotic controls. (D) Time series (in days) of the free copper concentration during growth of strain MD4 in presence or absence of CO, including abiotic or heat treated (autoclaved) controls (*n* = 3). (E) Electron microscope picture of strain MD4 culture after CO‐driven copper reduction. Scale bar size is 3 μm.

Conditions in halo‐alkaliphilic environments are often dynamic, resulting in fluctuating salinity and other element concentrations, which can additionally be driven by anthropogenic activity (Paul and Mormile 2017). To assess if strain MD4 was tolerant to increased levels of trace metals, the effect of copper, nickel and cobalt, was tested in concentrations ranging from 5 × 10^−3^ to 5 mM, with CO as a substrate (Figure 6). Of the tested metals, growth was halted by copper above 0.039 mM (Figure 6A), cobalt above 1.25 mM (Figure 6B) and nickel above 0.6 mM (Figure 6C). Interestingly, while strain MD4 did not grow in the presence of copper concentrations higher than 0.039 mM, a reddish colour was observed in the wells with > 1 mM copper. This was not observed in the abiotic control conditions or in any of the incubations with nickel or cobalt. This could point towards the reduction of the soluble Cu(II) ions to red coloured Cu(I) or Cu(0) precipitates, and the role of strain MD4 in this process was further studied in glass bottle tests (Figure 6D). Upon exposure of cells to 5 mM CuCl_2_ in the presence of CO, red precipitates formed within 1 day, lowering the free copper ion concentration to < 0.5 mM (Figure 6D). However, in contrast to observations in the 96‐well plate incubations, abiotic controls in glass bottles also exhibited red precipitate formation, albeit at a slower rate and less consistent between replicates (Figure 6D), suggesting abiotic reduction of copper by CO in the provided conditions. Incubations with heat‐treated (autoclaved) MD4 cells, as well as abiotic incubations containing the redox active molecule resazurin (0.1 g/L), in the presence of CO and copper led to the removal of solubilised copper(II) ions at a rate comparable to that observed with viable MD4 cells. In contrast, living cells in the absence of CO did not induce any changes in soluble copper(II) concentrations over time (Figure 6D). In both biotic and abiotic incubations, CO consumption and the decrease in soluble copper occurred at a molar ratio of 1:2, with no detectable formation or consumption of organic products, suggesting that Cu(II) is reduced by CO to Cu(I). Electron microscopy and energy‐dispersive x‐ray (EDX) analysis (Figure S3) confirmed the formation of spherical copper precipitates ranging in diameter from 1 to 2 μm (Figure 6E). Bacterial cells are in some cases observed to be partly encapsulated by the copper particles, but are mostly present outside the precipitates.

### 3.5 Functional Genomics and Transcriptomics

The genome of strain MD4 (Genbank: CP174120) has a size of 2.4 Mbp, with a GC content of 38.4%. The saccharolytic capabilities of the strain are confirmed from the genome, encoding the full glycolysis (Embden‐Meyerhof‐Parnas pathway) and the pentose phosphate pathway, enabling utilisation of hexose, as well as pentose sugars. From the pyruvate node, the central metabolism is connected to either the TCA cycle via pyruvate carboxylase, or to the acetyl‐CoA node via pyruvate‐formate lyase/pyruvate ferredoxin oxidoreductase. The TCA cycle is incomplete, lacking annotated genes encoding malate dehydrogenase and succinyl‐CoA synthetase. Most of the amino acids synthesis pathways were identified in the genome, with the exception of those for lysine, tyrosine and phenylalanine. The genome encodes pathways for the production of betaine and the synthesis of amino acids glutamate, glycine and proline that can potentially serve as osmolytes in high salt conditions (Sleator and Hill 2002).

**FIGURE 7:**
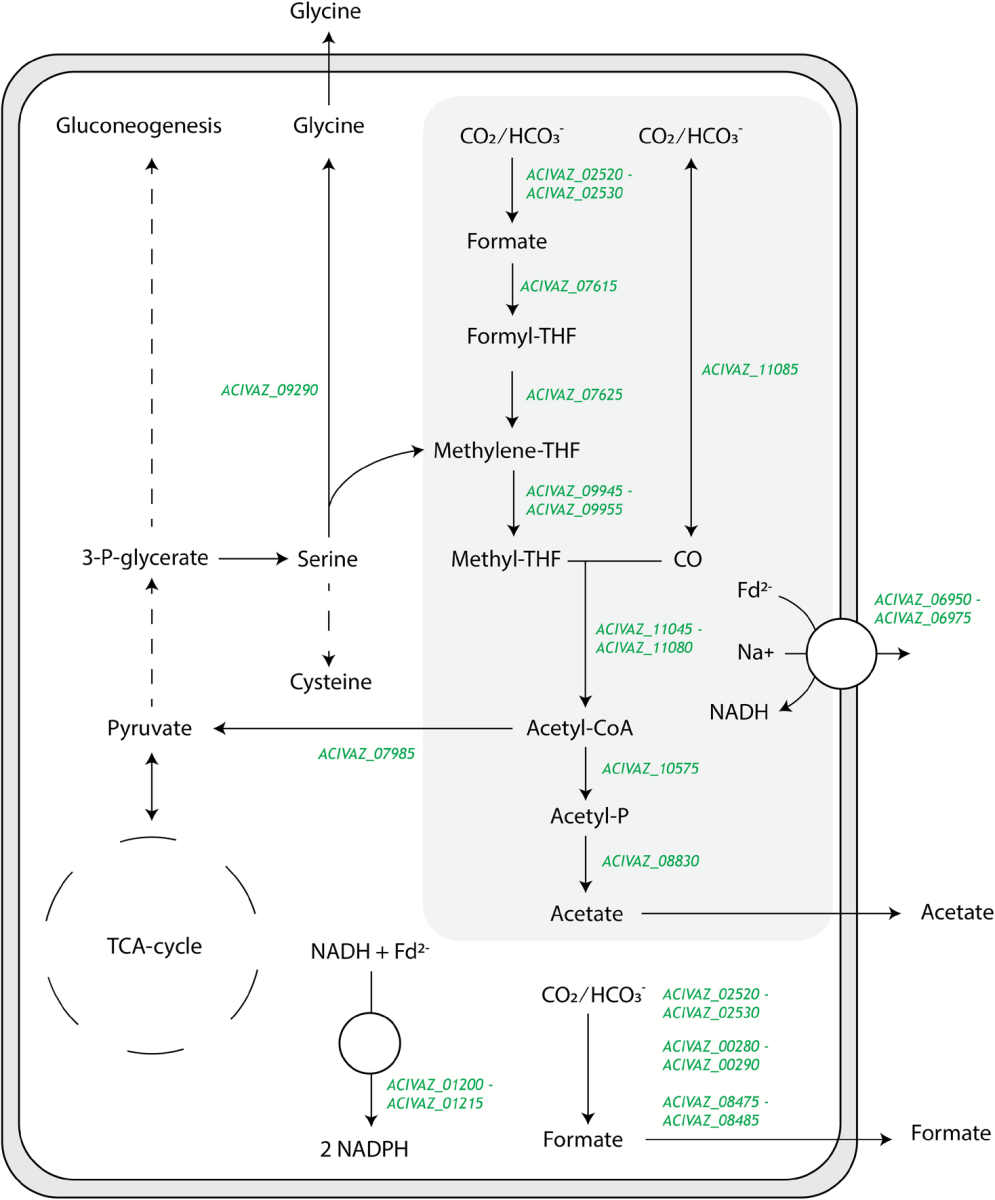
Schematic overview of the central metabolism of strain MD4, including gene numbers. The central Wood‐Ljungdahl pathway is represented by the grey shaded box. Dashed arrows indicate condensation of multiple reactions into one arrow.

For carboxydotrophic growth, all genes for the Wood‐Ljungdahl pathway (WLP) are present (Table 2, Figure 7), but are localized scattered across the genome. One large CODH operon was identified, carrying all subunits of the CODH/ACS complex, and associated proteins such as ferredoxins and maturation proteins (Table 2). Genes encoding a NADH‐dependent methylene‐THF reductase are located adjacent to an RnfC subunit, similar to the one identified in *A. woodii* (Bertsch et al. 2015). Additionally, the strain carries a predicted Na^+^‐dependent RnF complex (ACIVAZ_06950‐ACIVAZ_06975). The genome encodes an acetate kinase and potential phosphoacetyl‐transferase, fully completing the WLP for acetogenesis (Table 2). For formate production/consumption, next to pyruvate‐formate lyase, one NAD‐dependent formate dehydrogenase cluster was annotated in the genome (Table 2). This is unusual, as acetogens usually involve a bifurcating formate‐hydrogenase (Wang, Huang, Kahnt, Mueller, et al. 2013) or a formate‐hydrogen lyase (Schuchmann and Müller 2013) in the first step of the methyl‐branch of the WLP. Subunits for potential bifurcation activity are however not detected in the vicinity of the annotated NAD‐dependent formate dehydrogenase in strain MD4. The genome contains five gene clusters that show similarity to bifurcating hydrogenase/oxidoreductase complexes (Table 2), though a clear role in carboxydotrophic growth could not be assigned from genomic analysis alone.

**TABLE 2:** Overview of expressed genes involved in carboxydotrophic acetogenesis of strain MD4 via transcriptome analysis (*n* = 3), including genes from the WLP, energy‐conservation processes (bifurcation enzymes), and several relevant assimilatory reactions.

Expected function	GenBank id.	Annotation	Average counts ± stdev (×1000)	% of total counts
Formate dehydrogenase	ACIVAZ_02520	Formate dehydrogenase FdhF	40 ± 10	0.23
	ACIVAZ_02525	Molybdenum cofactor guanylyltransferase	3.4 ± 0.37	0.02
	ACIVAZ_02530	Formate dehydrogenase FdhD	4.8 ± 0.18	0.03
Formate‐THF ligase	ACIVAZ_07615	Formate‐THF ligase	69 ± 15	0.40
Methenyl‐THF cyclohydrolase	ACIVAZ_07625	5,10‐methenylTHF cyclohydrolase	28 ± 5.3	0.16
Methyl‐THF reductase	ACIVAZ_09945	Methylene‐THF reductase	23 ± 4	0.13
	ACIVAZ_09950	Methylene‐THF reductase	36 ± 8	0.21
	ACIVAZ_09955	Electron transport complex RsxC	81 ± 19	0.48
CODH/ACS	ACIVAZ_11045	Corrinoid activation protein AcsV	20 ± 5	0.12
	ACIVAZ_11050	ACS complex subunit alpha/beta subunit	79 ± 30	0.46
	ACIVAZ_11055	CODH/ACS methytransferase subunit	36 ± 17	0.21
	ACIVAZ_11060	ACS subunit gamma	80 ± 30	0.48
	ACIVAZ_11065	ACS subunit delta	15 ± 7	0.09
	ACIVAZ_11070	AAA family ATPase	17 ± 7	0.10
	ACIVAZ_11075	ACS subunit alpha/beta	260 ± 160	1,56
	ACIVAZ_11080	AAA family ATPase	70 ± 41	0.41
	ACIVAZ_11085	CODH catalytic subunit	250 ± 130	1,48
Acetyl‐transferase	ACIVAZ_10575	Bifunctional enoyl‐CoA hydratase/phosphate	15 ± 3	0.09
Acetate kinase	ACIVAZ_08830	Acetate kinase	100 ± 15	0.6
Potential bifurcating hydrogenase	ACIVAZ_00280	NAD(P)H‐dependent oxidoreductase subunit E	58 ± 13	0.34
	ACIVAZ_00285	NADH‐quinone oxidoreductase subunit F	76 ± 20	0.45
	ACIVAZ_00290	NADH‐dependent [Fe] hydrogenase	100 ± 33	0.61
Potential bifurcating hydrogenase	ACIVAZ_08475	NADH‐dependent [Fe] hydrogenase	67 ± 17	0.39
	ACIVAZ_08480	NADH–ubiquinone oxidoreductase‐F	59 ± 22	0.35
	ACIVAZ_08485	NADH‐quinone oxidoreductase subunit NuoE	18 ± 7	0.10
Potential Stn/nfn‐Complex	ACIVAZ_01200	Molybdopterin‐dependent oxidoreductase	25 ± 2.5	0.15
	ACIVAZ_01205	NADH‐quinone oxidoreductase subunit NuoF	5.6 ± 0.27	0.03
	ACIVAZ_01215	NADH‐quinone oxidoreductase subunit NuoE	0.5 ± 0.05	0.00
Potential bifurcating complex	ACIVAZ_06350	NAD(P)/FAD‐dependent oxidoreductase	6.7 ± 0.60	0.04
	ACIVAZ_06355	Molybdopterin‐dependent oxidoreductase	19 ± 0.40	0.11
	ACIVAZ_06360	4Fe‐4S dicluster domain‐containing protein	4.3 ± 0.30	0.03
Potential bifurcating complex	ACIVAZ_05780	Hydrogenase nickel incorporation hypB	0.15 ± 0.04	0.00
	ACIVAZ_05785	Hypothetical protein	0.64 ± 0.10	0.00
	ACIVAZ_05790	FAD‐dependent oxidoreductase	1.4 ± 0.40	0.01
Notable enzymes involved in assimilation	ACIVAZ_04225	Pyruvate carboxylase	95 ± 6.0	0.56
	ACIVAZ_07985	Pyruvate: ferredoxin oxidoreductase	45 ± 2.6	0.27
	ACIVAZ_02995	Pyruvate kinase	14 ± 2.8	0.09
	ACIVAZ_02515	Cysteïne synthase	240 ± 180	1,39
	ACIVAZ_09290	Serine hydroxymethyl transferase	0.9 ± 0.10	0.01

*Note:* Displayed counts (x1000) are averaged over 3 datasets and with standard deviation.

To confirm the genes involved in carboxydotrophic growth (Table 2), and in an attempt to identify the relevant genes involved in formate production and electron bifurcation activity, the transcriptome of CO‐grown strain MD4 was analyzed. As expected, genes involved in the WLP as well as CODH‐related genes are among the highest transcribed genes (Table 2). The NAD‐dependent formate dehydrogenase is clearly transcribed, suggesting a role during carboxydotrophic growth. Out of the five genomically identified putative bifurcating enzymes, two are highly abundant in the transcriptome (ACIVAZ_00290 and ACIVAZ_08475) and have an identical operon structure, consisting of three genes: an iron‐hydrogenase subunit, a NuoE‐like and NuoF‐like subunit. Furthermore, a molybdopterin‐dependent oxidoreductase (ACIVAZ_01200) is moderately expressed together with adjacent NuoE/F subunits. This complex shows similarity to the stnABC complex found in *Sporomusa ovata* (Kremp et al. 2020), and is thus potentially involved in the bifurcational reduction of NADP, using NADH and ferredoxin. The remaining two annotated oxidoreductases are: (I) a moderately expressed molybdopterin‐dependent oxidoreductase, flanked by oxidoreductase and ferredoxin‐like genes (ACIVAZ_06355) and (II) a weakly expressed FAD‐dependent oxidoreductase (ACIVAZ_05790).

For assimilation of acetyl‐CoA, it is likely that pyruvate ferredoxin oxidoreductase is used, rather than pyruvate formate lyase, due to its significantly higher expression (Table 2). Additionally, pyruvate kinase and pyruvate carboxylase are significantly expressed to connect the pyruvate node to gluconeogenesis and the partial TCA cycle, respectively (Table 2, Figure 7). Among some of the highest expressed genes during carboxydotrophic growth is the gluconeogenic pathway up to 3P‐glycerate, and subsequently the assimilation branch towards serine, glycine and cysteine (Table 2, Figure 7). Additionally, a glycine/betaine transport system (ACIVAZ_05010) is significantly expressed, suggesting potential import/export of these compounds during growth. Cysteine synthase (ACIVAZ_02515) is highly expressed, suggesting conversion of serine into cysteine. Finally, the highest expressed gene in the transcriptome is a catalase (ACIVAZ_06290), an indication of redox stress metabolism.

## 4 Discussion

Most characterised carboxydotrophic acetogens thrive in neutral or mildly acidic environments (pH 5–6), and were mostly isolated from gut or freshwater environments (Diender et al. 2015; Poehlein et al. 2025). When considering alkaline, high salinity environments, only two bacteria that can grow acetogenically on CO have been isolated—*Alkalibacter mobilis* (Khomyakova et al. 2021) and *Natranaerofaba carboxydivora* (Sorokin et al. 2021) – but have only been limitedly studied for their carboxydotrophic physiology. With the isolation and characterisation of strain MD4 we aimed to gain more insight into carboxydotrophic acetogenesis under these high salt and alkaline conditions.

Due to the strong negative redox potential of CO (E^0^’(CO/CO_2_) = −520 mV), its oxidation can be directly coupled to the reduction of ferredoxin (E^0^’ ~ −400 mV), posing a redox homeostasis challenge for carboxydotrophs. This challenge is often solved in acetogens by producing an ‘overflow’ of reduced products next to acetate (e.g., ethanol) (Allaart et al. 2023). During carboxydotrophic growth of strain MD4, ethanol production was not observed, but formate was a significant product (Figure 2). In alkaline conditions, proton concentration and protonated acids are scarce. Even though external pH may not directly reflect intracellular pH, some microbes are known to increase intracellular pH in high pH environments (Cook et al. 1996). This makes it possible that the intracellular pH of MD4 is more alkaline than that of acidophilic or neutrophilic acetogens, affecting the feasibility of reactions. Looking at the half‐reactions for proton reduction to hydrogen (Equation (1)), HCO_3_^−^ reduction to formate (Equation (2)) and acetate reduction to ethanol via acetaldehyde (Equations (3) and (4)), it becomes visible that ethanol production from acetate, and hydrogen production both strongly relate to proton concentration, making them potentially energetically poor redox homeostasis mechanisms at elevated pH. Production of formate also depends on consumption of protons, but in carbonated alkaliphilic systems this is partly compensated for at increased concentrations of bicarbonate.

(1) $${\text{2e}}^{-}+{\text{2H}}^{+}\to H_{2}\;\left({E^{0}}^{’}=-414\;\mathrm{mV}\right)$$

(2) $${{\mathrm{HCO}}_{3}}^{-}+2\;H^{+}+{\text{2e}}^{-}\to {{\mathrm{HCO}}_{2}}^{-}+H_{2}O\;\left({E^{0}}^{’}=-430\;\mathrm{mV}\right)$$

(3) $${\mathrm{CH}}_{3}{\mathrm{COO}}^{-}+3\;H^{+}+2\;e^{-}\to {\mathrm{CH}}_{3}\mathrm{CHO}+H_{2}O\;\left({E^{0}}^{’}=-580\;\mathrm{mV}\right)$$

(4) $${\mathrm{CH}}_{3}\mathrm{CHO}+2\;H^{+}+2\;e^{-}\to C_{2}H_{5}\mathrm{OH}\;\left({E^{0}}^{’}=-197\;\mathrm{mV}\right)$$

Growth of strain MD4 showed a positive relation between elevated carbonate concentration and production of formate (Figure 5), suggesting a role for bicarbonate in the process. It remains unclear whether (bi)carbonate can act directly as a substrate, or if it must first be converted to CO_2_ before serving as an electron acceptor, as observed for other formate dehydrogenases (Rusching et al. 1976). Under continuous CO supply, a high formate:acetate production ratio was observed (Figure 2B), suggesting that formate production serves as a redox homeostasis mechanism during growth at elevated CO concentrations. This is additionally confirmed by the formate production profiles at high and low CO pressures (Figure 3), where formate becomes a more significant product at higher CO pressures (Figure 3). Moreover, the initial presence of formate at high CO pressures appears to inhibit growth, indicating that the formation of formate is thermodynamically or kinetically constrained. Formate production from CO is likely linked to energy conservation, with the RnF‐complex providing NADH for formate dehydrogenase activity—regardless of whether this complex functions bifurcatively (Figure S4). This explains the growth of the culture despite minimal acetate pr in the reactor (~1.5 mM). In bottles, when CO pressure is low, formate is partially co‐consumed with residual CO, contributing to acetate formation (Figures 2A and 3). This behaviour resembles ethanol re‐consumption during carboxydotrophic growth of *C. autoethanogenum*, where ethanol serves as temporary storage of energy and redox equivalents (Diender et al. 2023). Transient formate production during carboxydotrophic growth of strain MD4 could thus be an alternative form of carboxydotrophic overflow metabolism (Allaart et al. 2023), used by acetogens in alkaline conditions. Formate production is often observed in alkaliphilic systems (Garnova et al. 2004; Namirimu et al. 2022; Sorokin et al. 2011, 2021; Sousa et al. 2015), where it thus potentially serves as a form of redox homeostasis mechanism.

Strain MD4 utilises the Wood‐Ljundahl pathway for carboxydotrophic acetogenesis (Figure 7, Table 2). Despite the significant production of formate (Figures 2 and 3), known bifurcating formate dehydrogenases, or formate hydrogen‐lyases, could not be identified from its genome. The sole annotated formate dehydrogenase (ACIVAZ_02520) is annotated as NADH‐dependent, but shows some similarity with the bifurcating FdhF2 from *Gottschalkia acidurici* (Wang, Huang, Kahnt, and Thauer 2013). In *G. acidurici*, the formate dehydrogenase is associated with a bifurcating iron‐hydrogenase, catalysing the bifurcation of NADH and ferredoxin to reduce CO_2_ to formate. Similar bifurcating formate dehydrogenases are found in other acetogens like *C. autoethanogenum* and *Clostridium ljungdahlii* (Wang, Huang, Kahnt, Mueller, et al. 2013). The formate dehydrogenase in strain MD4 is not genomically located close to a hydrogenase, making predictions on its bifurcating properties difficult. In the transcriptome, two potential bifurcating iron‐hydrogenases are highly expressed during carboxydotrophic growth (Table 2), and are both located next to co‐expressed Nuo E/F subunits, often associated with bifurcating hydrogenases (Katsyv et al. 2023; Sazanov and Hinchliffe 2006). Recently, the structure and bifurcation mechanism of these types of hydrogenases (HydABC) in *Acetobacterium woodii* have been elucidated, showing how H_2_ oxidation is linked to ferredoxin and NAD(P) reduction (Katsyv et al. 2023). The production of formate using only NADH is generally considered to be thermodynamically poorly favourable (Soboh et al. 2004), but on the other hand, the high bicarbonate availability might promote the feasibility of formate production, making its production potentially not dependent on a bifurcating formate dehydrogenase. However, since no hydrogen production or consumption is observed in strain MD4, the two highly expressed hydrogenase complexes, together with the identified NAD‐dependent formate dehydrogenase, could potentially form a bifurcating formate dehydrogenase that drives the observed formate production, as well as catalyse the first step of the WLP. Further in‐depth biochemical experiments are however required to elucidate the exact function of these enzymes.

The transcriptome data also showed high expression of the pathway towards 3P‐glycerate and serine. As genes for betaine synthesis are not clearly expressed, serine could be converted into glycine via the present glycine hydroxymethyltransferase (ACIVAZ_09290). This is supported by the detection of glycine in the growth medium, while other amino acids were not detected. Potential use of the reductive glycine pathway to support CO_2_ fixation as shown in *Clostridium drakei* (Song et al. 2020) is unlikely as no glycine reductase could be detected in the genome of strain MD4. Additionally, high expression of cysteine synthase suggests cysteine production is relevant, but as titanium‐citrate (instead of sulfide) was used as a reducing agent in the transcriptome experiment, its upregulation might be related to promoting sulfur assimilation metabolism.

The source of isolation of strain MD4 was a syngas‐fed bioreactor, operated at high salt and alkaline conditions, and was originally inoculated with a mix from various saline sources (Sousa et al. 2015). Due to this mixed inoculum, followed by multiple months of enrichment of the microbial community during bioreactor operation, the exact environmental origin of strain MD4 remains unresolved. Additionally, 16S rRNA gene analysis on the bioreactor samples, even after extended operation with CO in the feed gas, did not identify any members of the *Alkalibacteraceae* family (Plugge et al. 2020; Sousa et al. 2015) suggesting low abundance of strain MD4 in the original bioreactor sample (but could alternatively be due to methodological limitations in DNA extraction and amplification). Strain MD4 was able to grow in a wide range of pH, bicarbonate and sodium concentrations (Table 1, Figure 4), indicating a high degree of physiological adaptability and osmotic tolerance. This trait is consistent with conditions found in habitats such as salt ponds, or environments that are subject to evaporation events. Given this adaptability, we also tested potential elevated resistance of strain MD4 to trace metals (nickel, cobalt, and copper) (Figure 6). Described toxicity limits in prokaryotes for copper range widely from 10^−8^ to 10^−3^ M (Zevenhuizen et al. 1979), while toxicity studies in sludge samples with nickel and cobalt indicate toxicity effects starting around 0.5 × 10^−3^ M (Gikas 2008). The metal concentrations found detrimental for strain MD4 do not strongly exceed these values, making the bacterium not exceptionally adapted to elevated trace metal concentrations. The observed CO‐driven reduction of copper in several cultures of strain MD4, seems abiotic due to the observation of similar reduction patterns with dead cells and in abiotic controls (Figure 5D). The copper reduction rate, however, appears stimulated by the presence of bacterial cells or redox active molecules like resazurin, suggesting a speculative catalytic role of cells and/or redox active molecules in the observed CO‐driven copper reduction. Additionally, the electron microscopic observations show that some bacterial cells are encapsulated by copper (Figure 6E), indicating that cells potentially function as a surface where the copper precipitates.

Based on whole genome taxonomy, MD4 clusters within the recently proposed *Alkalibacteraceae* family (Chuvochina et al. 2023), forming a distinct branch between the *Alkalibacter* and *Alkalibaculum* genera (Figure 1). The closest characterised isolate to strain MD4 is *Alkalibacter mobilis*, with a 16S rRNA gene identity of 92.6%. This identity is well below the posed cutoff for novel genera of < 95% (Konstantinidis et al. 2017). Its novelty is further supported by the dDDH analysis, showing a highest parameter d_4_ value of 36.3% against *A. bacchi* , reflecting low similarity in the homologous part of the compared genomes. The AAI analysis shows similarity values of strain MD4 of around 70% to members of the Alkalibacter genus, being on the border of the cutoff value for novel genera (< 65%). However, the coverage of the total proteome between the members of the Alkalibacter genus and strain MD4 for AAI analysis is only ~25%, whereas on a genus level a coverage of at least 50% is expected (Riesco and Trujillo 2024). Therefore, based on the combination of 16S rRNA gene, dDDH, and AAI analyses we propose that strain MD4 is the first representative of a novel genus.

## 5 Taxonomy

Based on the phylogenomic analysis and observed phenotypic properties, we propose to classify strain MD4 into a new genus and species *Haloacetibacter carboxydivorans*, placing it as a member of the family *Alkalibacteraceae*.

### 5.1 Haloacetibacter Gen. Nov

Ha.lo.a.ce.ti.bac'ter. Gr. masc. n. hals, salt; L. neut. n. acetum, vinegar; N.L. masc. n. bacter, rod; N.L. masc. n. Haloacetibacter, an acetate‐producing rod from a salty environment.

The genus includes anaerobic acetogenic bacteria with rod‐shaped cells. They are moderately halophilic and alkaliphilic and grow at mesophilic temperatures. They can perform fermentative acetogenic growth on sugars or on CO. Fermentation products mainly include acetate and formate. *Haloacetibacter* forms a branch within the *Alkalibacteraceae*. The type species is *Haloacetibacter carboxydivorans*.

### 5.2 Haloacetibacter Carboxydivorans sp. Nov

Car.bo.xy.di.vo'rans L. pref. carboxydum, carbon monoxide; L. v. vorare, to devour, consume; L. part. adj. carboxydivorans, consuming carbon monoxide.

Cells are rod‐shaped, 1–5 μm long, occasionally occurring in chains of multiple cells and staining Gram‐positive. Membrane lipid composition consists mainly of a variety of C16 and C18 ester‐bound alkyl chains, and includes a significant amount of plasmalogen lipids. The species grows acetogenically on sugars or carbon monoxide, producing acetate and formate as main fermentation products. Respiratory metabolism was not observed. It grows moderately alkaliphilic between a pH of 7 and 10 (opt. 8–9), halophilic at 0.01–2.5 M sodium (opt. 0.8–1.2 M) and at mesophilic temperatures of 15°C–40°C (opt. 35°C–37°C). The presence of carbonate was required for growth. The type strain was enriched and isolated from biomass samples collected from a syngas CO‐fed bioreactor operated at haloalkaline conditions. The type strain is MD4^T^ DSM 119686 and the genome is deposited in GenBank accession number CP174120.

## Author Contributions

**Martijn Diender:** conceptualization, investigation, funding acquisition, writing – original draft, methodology, visualization, supervision, formal analysis, writing – review and editing. **Isabelle M. Keijsers:** methodology, validation, investigation, writing – review and editing. **Anastasia Galani:** formal analysis, visualization, writing – review and editing, investigation. **Timo van Roosmalen:** validation, investigation, methodology. **Alfons J. M. Stams:** writing – review and editing, funding acquisition, supervision. **Diana Z. Sousa:** writing – review and editing, funding acquisition, supervision.

## Funding

This work was supported by Nederlandse Organisatie voor Wetenschappelijk Onderzoek (VI.VENI.212.112, OCENW.XS21.1.023, P16‐10, project 1) and the Dutch Ministry of Education and Science (Project 024.002.002: Soehngen Institute of Anaerobic Microbiology).

## Conflicts of Interest

The authors declare no conflicts of interest.

## Supporting information


**Data S1:** Supporting Information

**Table S1:** Membrane lipid composition of strain MD4 grown on glucose.
**Figure S1:** AAI values of the proteome of strain MD4. Total matching proteome fraction is shown on the Y‐axis, while average identity values are displayed on the X‐axis.
**Figure S2:** Temperature (a) and pH (b) profile of strain MD4 when grown on glucose as a substrate. The increase in OD600 above 45°C and pH of 10 was disturbed due to colorization of the medium as a result of Maillard reactions between the yeast extract and the glucose.
**Figure S3:** Scanning electron microscope (SEM) and EDX analysis on copper particles formed in presence of strain MD4 bacterial cells. Samples were Tungsten coated, causing the assigned peaks for Tungsten (W) and Yttrium (Y). Figure panels show: (A) SEM overview image, (B) EDX analysis spectrum of point 2 (Copper particle), (C) EDX analysis spectrum of point 3 (MD4 bacterial cell), (D) EDX analysis spectrum of point 7 MD4 bacterial cell.
**Figure S4:** Proposed mechanism for energy conservation during carboxydotrophic formate production in strain MD4 using either (A) a bifurcating formate dehydrogenase or (B) a NADH‐dependent formate dehydrogenase.

## Data Availability

The data that supports the findings of this study is available in the Supporting Information of this article, and can be found in relevant databases further specified in material and methods.
