# Supplementary material for: Carboxydotrophic Acetogenesis in Alkaline Conditions Results in Transient Formate Production by the Halo‐Alkaliphilic Acetogen Haloacetibacter carboxydivorans Gen. Nov. sp. Nov
Source: Environ Microbiol Rep. 2026 Jan 27;18(1):e70254. doi: 10.1111/1758-2229.70254 (PMC12835891; doi:10.1111/1758-2229.70254)

**Supplementary table 1. Membrane lipid composition of strain MD4 grown on glucose.**

| Carbon number | Lipid | Presence (%) | Summed abundance (%) |
| --- | --- | --- | --- |
| 12 | 12:0 FA | 1.28 | 1.28 |
| 14 | 14:0 FA | 16.61 | 16.61 |
| 15 | 15:2 FA | 1.26 | 1.26 |
| 16 | 16:0 ALDE | 2.36 | 44.56 |
|  | 16:1 CIS 7 FA | 2.24 |  |
|  | 16.1 CIS 9 FA | 6.53 |  |
|  | 16:0 FA | 16.04 |  |
|  | 16:1 CIS 7 DMA | 1.79 |  |
|  | 16:1 CIS 9 DMA | 2.67 |  |
|  | 16:0 DMA | 12.93 |  |
| 17 | 17:2 FA | 1.08 | 1.08 |
| 18 | 18:0 ALDE | 0.76 | 35.22 |
|  | 18:1 CIS 9 FA | 3.21 |  |
|  | 18:1 FA | 7.76 |  |
|  | 18:0 FA | 7.18 |  |
|  | 18.1 CIS 9 DMA | 4.51 |  |
|  | 18:1 CIS 11 DMA | 7.38 |  |
|  | 18:0 DMA | 4.42 |  |
|  |  |  |  |
|  | total |  | 100.01 |
| FA = Fatty acid, derived from ester-bound alkyl chains  DMA = Dimethyl acetals, derived from alk-1-enyl ether substituents of plasmalogen lipids | | | |

**Supplementary figure S1.** AAI values of the proteome of strain MD4. Total matching proteome fraction is shown on the Y-axis, while average identity values are displayed on the X-axis.


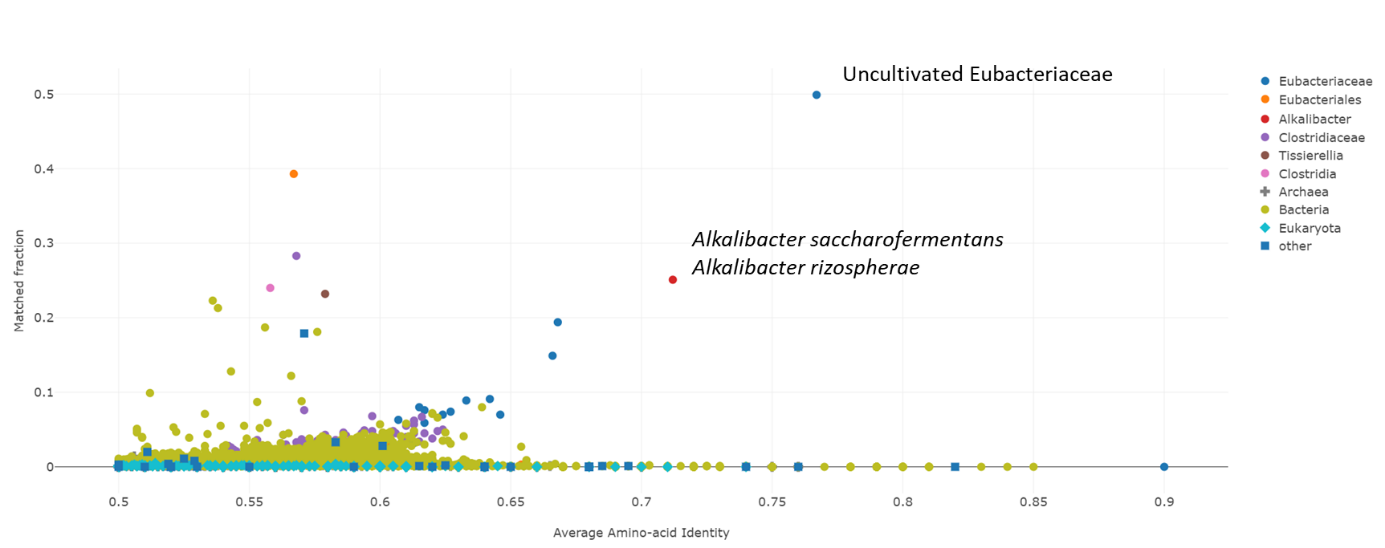


**Supplementary figure S2.** Temperature (a) and pH (b) profile of strain MD4 when grown on glucose as a substrate. The increase in OD600 above 45 ^○^C and pH of 10 was disturbed due to colorization of the medium as a result of Maillard reactions between the yeast extract and the glucose.


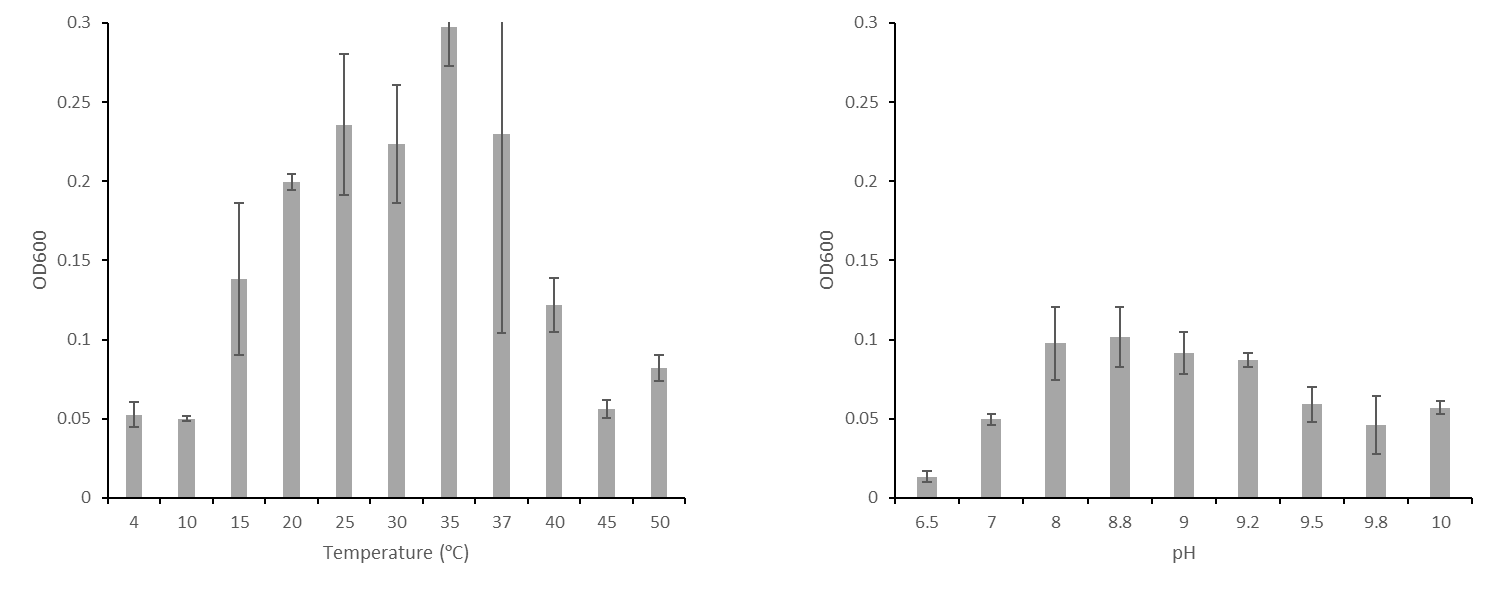


**
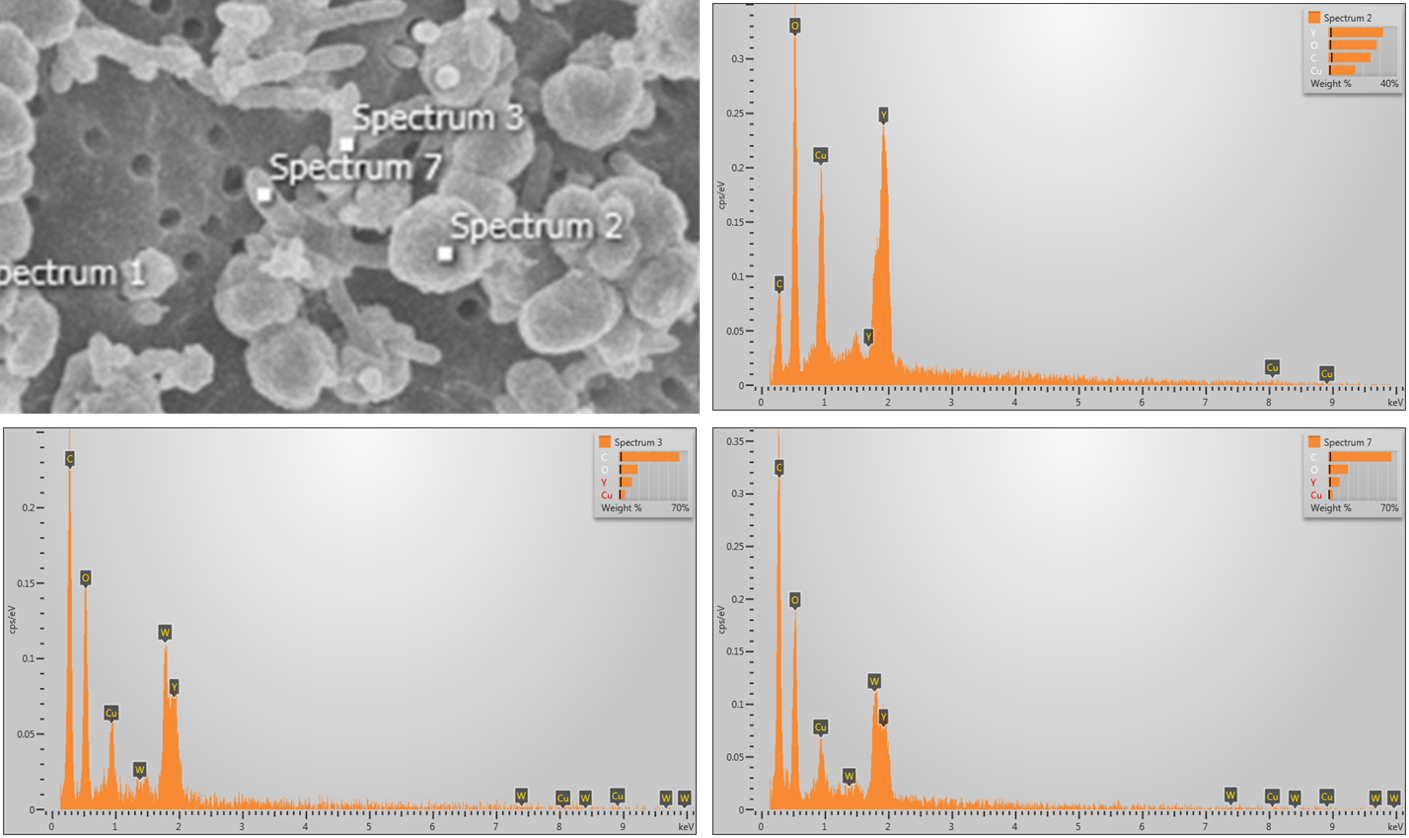
Supplementary figure S3.** Scanning electron microscope (SEM) and EDX analysis on copper particles formed in presence of strain MD4 bacterial cells. Samples were Tungsten coated, causing the assigned peaks for Tungsten (W) and Yttrium (Y). Figure panels show: A) SEM overview image, B) EDX analysis spectrum of point 2 (Copper particle), C) EDX analysis spectrum of point 3 (MD4 bacterial cell), D) EDX analysis spectrum of point 7 MD4 bacterial cell).

**Supplementary figure S4.** Proposed mechanism for energy conservation during carboxydotrophic formate production in strain MD4 using either A) a bifurcating formate dehydrogenase or B) a NADH-dependent formate dehydrogenase.


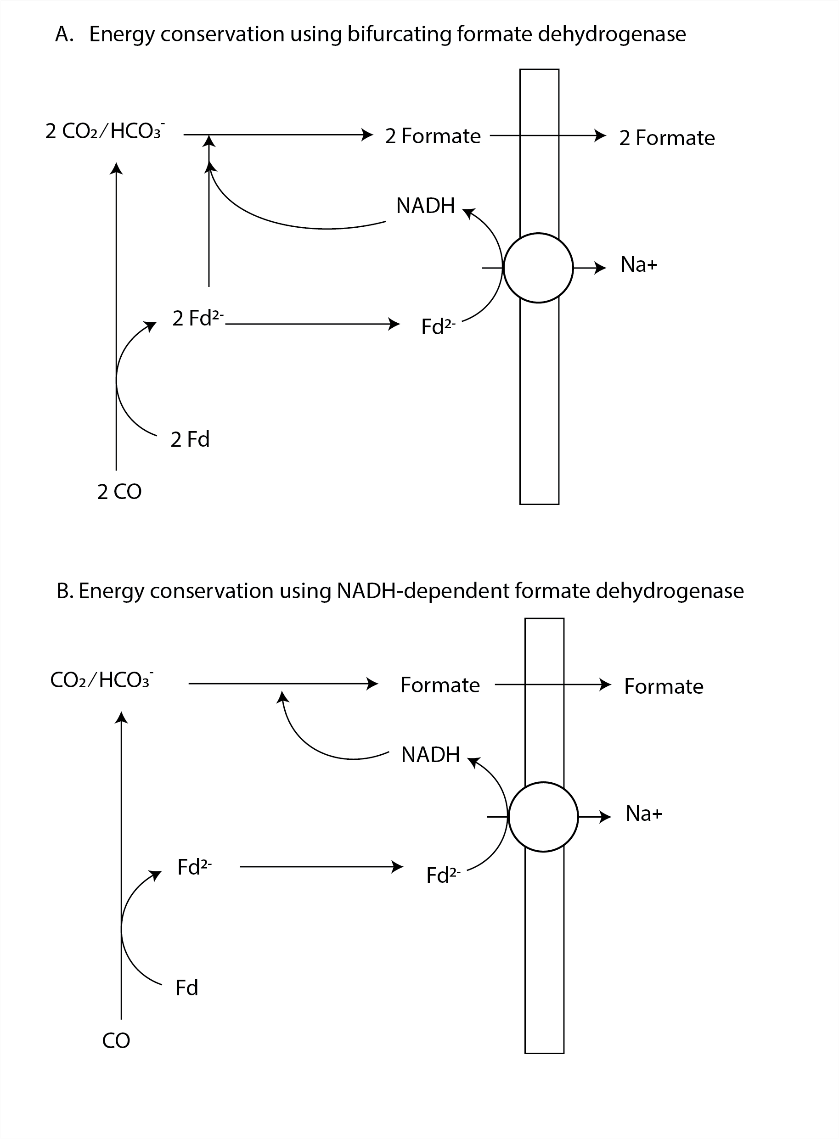

Supplement: Supplementary file 2 — Table S1: Membrane lipid composition of strain MD4 grown on glucose. Figure S1: AAI values of the proteome of strain MD4. Total matching proteome fraction is shown on the Y‐axis, while average identity values are displayed on the X‐axis. Figure S2: Temperature (a) and pH (b) profile of strain MD4 when grown on glucose as a substrate. The increase in OD600 above 45°C and pH of 10 was disturbed due to colorization of the medium as a result of Maillard reactions between the yeast extract and the glucose. Figure S3: Scanning electron microscope (SEM) and EDX analysis on copper particles formed in presence of strain MD4 bacterial cells. Samples were Tungsten coated, causing the assigned peaks for Tungsten (W) and Yttrium (Y). Figure panels show: (A) SEM overview image, (B) EDX analysis spectrum of point 2 (Copper particle), (C) EDX analysis spectrum of point 3 (MD4 bacterial cell), (D) EDX analysis spectrum of point 7 MD4 bacterial cell. Figure S4: Proposed mechanism for energy conservation during carboxydotrophic formate production in strain MD4 using either (A) a bifurcating formate dehydrogenase or (B) a NADH‐dependent formate dehydrogenase. [file EMI4-18-e70254-s002.docx]
